# Electronic collection of patient-reported outcomes following discharge after surgery: systematic review

**DOI:** 10.1093/bjsopen/zraa072

**Published:** 2021-03-30

**Authors:** C Tsang, K S Lee, H Richards, J M Blazeby, K N L Avery

**Affiliations:** 1 Bristol Centre for Surgical Research, Bristol Medical School: Population Health Sciences, University of Bristol, Bristol, UK; 2 Bristol Medical School, University of Bristol, Bristol, UK; 3 Division of Surgery, Head and Neck, University Hospitals Bristol and Weston NHS Foundation Trust, Bristol, UK

## Abstract

**Background:**

Little is known about the electronic collection and clinical feedback of patient-reported outcomes (ePROs) following surgical discharge. This systematic review summarized the evidence on the collection and uses of electronic systems to collect PROs after discharge from hospital after surgery.

**Method:**

Systematic searches of MEDLINE, Embase, PsycINFO, CINAHL and Cochrane Central were undertaken from database inception to July 2019 using terms for ‘patient reported outcomes’, ‘electronic’, ‘surgery’ and ‘at home’. Primary research of all study designs was included if they used electronic systems to collect PRO data in adults after hospital discharge following surgery. Data were collected on the settings, patient groups and specialties, ePRO systems (including features and functions), PRO data collected, and integration with health records.

**Results:**

Fourteen studies were included from 9474 records, including two RCTs and six orthopaedic surgery studies. Most studies (9 of 14) used commercial ePRO systems. Six reported types of electronic device were used: tablets or other portable devices (3 studies), smartphones (2), combination of smartphones, tablets, portable devices and computers (1). Systems had limited features and functions such as real-time clinical feedback (6 studies) and messaging service for patients with care teams (3). No study described ePRO system integration with electronic health records to support clinical feedback.

**Conclusion:**

There is limited reporting of ePRO systems in the surgical literature, and ePRO systems lack integration with hospital clinical systems. Future research should describe the ePRO system and ePRO questionnaires used, and challenges encountered during the study, to support efficient upscaling of ePRO systems using tried and tested approaches.

## Teaser

This systematic review examined the use of electronic systems to collect patient-reported outcomes (ePROs) post-discharge after surgery.

Fourteen studies were included - most used commercial ePRO systems, few reported the types of electronic devices used, systems had limited features and functions and none described integration of the system with electronic health records.

There is limited reporting of ePRO systems in surgical literature.

## Introduction

Routine electronic collection of patient-reported outcomes (ePROs) and the electronic communication (feedback) of these data to patients and clinicians through the ePRO system are associated with improved clinical outcomes and enhanced quality of life for patients^1–3^. Timely and easy access to ePRO feedback may enhance patient–clinician communication and information-sharing between patients and their care teams^4^. Additionally, prompt provision of advice and information to patients may support self-management of symptoms to enhance recovery after medical treatment^5^.

Most ePRO research has focused on the collection and application of data during outpatient appointments or hospital admissions. Typically, such studies have focused on patients receiving specific treatments, most notably those receiving chemotherapy^6–8^. The ePRO systems used in these studies vary considerably in terms of how frequently and where they are accessed (for instance, daily or every 3 months; at home or in hospital), the extent of integration with clinical processes (such as a standalone system with no connection to electronic medical records), and in the amount and timing of feedback they provide (for example, patients do not typically have access to submitted data)^6–8^.

Evidence for the use of ePRO systems in other patient groups, such as those undergoing surgery, is sparse. Patients who require a hospital stay for surgery may experience a wider range of, and more serious, complications after surgery than those who undergo surgery as a day case. With increasing use of enhanced recovery after surgery (ERAS) programmes to support quicker and better recovery^9–11^, patients are discharged sooner after surgery. However, they often receive limited and inconsistent follow-up care in the first days and weeks after their discharge home^12–14^. Therefore, during this critical recovery period, patients may benefit particularly from remote monitoring to detect postoperative complications and manage their symptoms^15^^,^^16^. To support the optimal delivery of safe, patient-centred care, ePRO data should be integrated with electronic health records (EHRs) and should be fed back in real-time to clinicians^17^. Yet it is unclear the extent to which ePRO systems have been successfully integrated with EHRs and how clinicians and patients engage with ePRO data during recovery at home after surgery. A systematic review was undertaken to examine the use of electronic systems to collect ePROs following discharge after surgery. The review specifically investigated the settings, patient groups, and specialties in which ePRO systems have been used. It also examined the ePRO systems, including their features and functionalities, patient-reported outcome (PRO) data collected by these systems, and the impact of ePRO systems on clinical or patient-reported outcomes.

## Methods

The review was conducted according to the PRISMA guidelines^18^^,^^19^, and was registered on PROSPERO (registration number CRD42019144806). Searches of the following five electronic databases were undertaken: Ovid MEDLINE, Ovid Embase, Ovid PsycINFO, Cumulative Index to Nursing and Allied Health Literature (CINAHL) and the Cochrane Central Register of Controlled Trials (CENTRAL). Searches were performed in each database from its inception until 11 July 2019. The search strategy was developed in consultation with a research librarian, based on the Consensus-based Standards for the selection of health Measurement Instruments (COSMIN) guideline for systematic reviews of patient-reported outcome measures and published reviews of ePRO systems^20–22^. The concepts of ‘patient reported outcomes’, ‘electronic’, ‘surgery’ and ‘at home’ were used in addition to synonyms and related terms. An example search strategy used for Ovid MEDLINE is presented in *Appendix S1*. Reference lists of publications included in the full review were searched to identify further studies for inclusion.

### Eligibility criteria

#### Types of study

Primary research studies on ePRO data collection were eligible for inclusion: randomized, quasi-experimental, and observational studies, including case series and case reports.

#### Study populations

The review included studies on adult patients aged 18 years or older who met all the following criteria: admitted to hospital; an inpatient stay of at least one night (not planned day cases); received surgery; and subsequent discharge. Studies on patients receiving more than one treatment were included if at least one treatment was surgical and was received during an inpatient stay.

#### Intervention/comparators

Studies on the use of electronic systems to collect PRO data (ePRO data) were included when at least some data were collected after discharge, outside the inpatient or outpatient healthcare settings (for instance in patients’ homes). Studies with ePRO data as primary or secondary outcome measures were eligible for study inclusion.

### Exclusion criteria

Studies were excluded if they reported the development or set-up of ePRO systems (also referred to as ‘feasibility studies’) but did not describe and report results for collected ePRO data. The review also excluded narrative and systematic reviews, editorials, commentaries, opinion papers, letters, education papers, conference abstracts, protocols, reports, theses or book chapters, as they were unlikely to contain sufficient detail about the features and functionality of individual ePRO systems. Articles in languages other than English were also excluded.

### Study selection

Titles and abstracts of articles were screened independently by two reviewers using the predefined eligibility criteria. Disagreements between the two reviewers were resolved by a third reviewer. Where abstracts were unavailable or the content inadequate to decide on eligibility, full articles were obtained and reviewed. Screening of articles, including removal of duplicates, was managed using EndNote™ X9 software (Clarivate Analytics, Philadelphia, PA, USA).

### Data extraction

Data on the following topics were extracted from full-text articles after screening: publication details; study design; patient, disease and treatment characteristics; study methods; ePRO system and its features; ethics and governance; and results. A data extraction template form was developed for the review to ensure standardization and consistency in this process. Extracted data were collated in Microsoft Excel^®^ (Microsoft, Redmond, WA, USA). Three reviewers independently extracted data, with additional review to confirm the accuracy and completeness of data extraction. Discrepancies or disagreements about extracted material were resolved discussion with another reviewer.

### Data synthesis

A narrative synthesis of data, with descriptive analyses where appropriate, was undertaken. Risk of bias in individual studies and across studies was not assessed as only descriptive analyses were undertaken in this study and quantitative synthesis techniques (such as meta-analysis) were not applied. A meta-analysis was not performed as the aim of this review was to identify and describe, rather than to establish, the effectiveness of, ePRO systems.

## Results

**Fig. 1:**
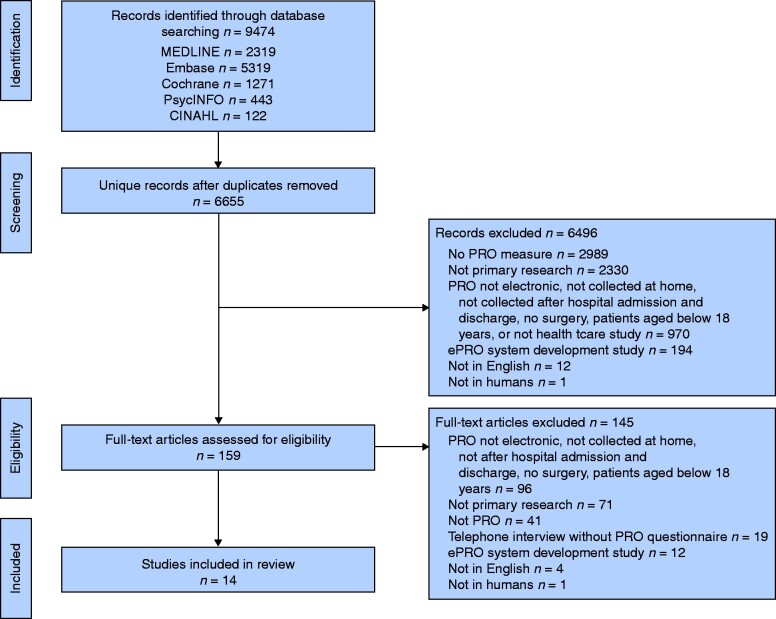
Flow diagram showing selection of studies for review *Multiple reasons for exclusion may apply. PRO, patient-reported outcome; ePRO, electronic system for collection of PROs.

A total of 9474 articles were retrieved. After excluding ineligible articles during initial screening, 159 publications were obtained for further eligibility assessment (*Fig. 1*). Of these, 145 (91.2 per cent) were excluded after full-text screening. The remaining 14 articles^16^^,^^23–35^ were included in the review (*Table S1*). These included studies were published between 2003 and 2019. Approximately half of studies (8 of 14) were conducted in the USA, and the others were done in Europe. There were two RCTs. Two studies were conducted at more than one site (10 sites in each study).

### Patient groups and surgical specialties

Across the 14 studies, a total of 2951 patients (ranging from 20 to 1076) participated and 2424 (ranging from 15 to 1076) completed follow-up. Almost half of the studies (6 of 14) were in orthopaedic populations, such as patients who received knee, hip or shoulder arthroplasty, and those who had an anterior cruciate ligament repair. Other patient groups included those who had colorectal cancer surgery (1 study), vascular surgery (1) or gynaecological surgery (1). The two RCTs studied the use of ePRO systems in patients undergoing surgery for vascular disease and colorectal cancer.

Where stated (13 studies), the length of patient follow-up ranged from 5 days to approximately 2 years. All but one study reported the method(s) used for patient follow-up. Almost half of the studies (6 of 14) used multiple methods that included at least two of the following: the ePRO system; e-mail; telephone; and face-to-face contact. The other studies reported the sole use of the ePRO system (6 studies) or only e-mail (1 study) for follow-up. In 10 studies the primary outcome measure was patient-reported, for example the European Organization for Research and Treatment of Cancer (EORTC) Core Quality of Life Questionnaire (QLQ-C30) version 3.0, Memorial Sloan Kettering Cancer Center Bowel Function Instrument (MSKCC BFI) and visual analogue scale (VAS) pain score. The remaining four studies used physical activity sensors for continuous or automated data collection as the primary outcome measure.

### Features and functionality of ePRO systems

In most studies (9 of 12) that described the ePRO system software, a commercial product was used. Two other studies reported the use of software developed in-house. The types of electronic device used to collect ePRO data were reported in six studies: tablet/portable device (3 studies); smartphone (2), and combinations of smartphone, tablet/portable device and computer (1).

None of the 14 studies reported that the ePRO system was integrated with EHRs. Feedback of ePRO data to clinicians was rare: six systems provided real-time feedback to clinicians and four provided delayed feedback (*Table 1*). Fewer studies reported the use of other ePRO features and functions, such as a messaging service for patients to communicate directly with their care team (3 studies), automated patient reminders to complete questionnaires or submit data (3), or graphical displays for patients to view reported symptoms (1).

**Table 1:** Features and functions of electronic systems used to collect patient-reported outcomes

Features and functions	**No. of studies**
Real-time feedback to clinicians	6
Delayed feedback to clinicians	4
Direct communication with clinical care team	3
Automated patient reminders	3
Patient diary or log	3
Real-time feedback or advice to patients	2
Graphical patient record of reported symptoms	1
Personal health record	1

This systematic review examined the use of electronic systems to collect patient-reported outcomes (ePROs) following discharge after surgery. Fourteen studies were included; most used commercial ePRO systems, few reported the types of electronic device used, systems had limited features and functions, and none described integration of the system with electronic health records.

There is limited reporting of ePRO systems in the surgical literature.

Four studies reported the use of ePRO data to inform patient care. In two of these studies, predefined criteria and corresponding thresholds for patient-reported data triggered automated notifications to the clinical care team. One of these studies reported that alerts were by e-mail, but the other study did not specify the mode in which alerts were received by the clinical team. In the other two studies, ePRO data were considered alongside clinical examination, but automated notifications were not sent.

### Patient-reported outcome data collected by ePRO systems

Almost all studies (12 of 14) collected more than one type of PRO data through the ePRO system. Postoperative symptoms and problems were most commonly collected (13 studies), such as pain and sleep disturbance. Few studies collected data on quality of life (4). Most studies (11) used at least one validated questionnaire to measure PROs. These included the MSKCC BFI, the Western Ontario Shoulder Instability Index (WOSI), and modified instruments such as a single item adapted from the Subjective Significance Questionnaire (SSQ). The three other studies reported the use of diaries (1 study) and questionnaires (2 studies) developed specifically for the study, with no information on whether these instruments were validated before use in the respective studies. Where formats in which PRO data were collected were described, these ranged from VAS ratings (3 of 12); ordinal scoring including Likert scales (11 of 12) and simple count data (7 of 12). The frequency of data collection using the ePRO systems varied, ranging from a single time point to annually. Six studies collected data once or twice a day. Studies that included the collection of physical activity data reported that data were collected and uploaded passively, either continuously and/or automatically (5 studies).

### Impact of ePRO systems on clinical or patient-reported outcomes

Approximately half of studies (7 of 14) collected data on patients’ satisfaction with the electronic reporting system. One study assessed the economic impact of digital rehabilitation (including ePRO) compared with home visits from healthcare professionals in addition to digital rehabilitation after total hip arthroplasty. It found that the use of only electronic applications to support rehabilitation, including ePROs, was clinically non-inferior to digital rehabilitation in addition to home visits, but was significantly cheaper. No other study explicitly considered whether or how ePRO systems affected clinical or patient-reported outcomes.

## Discussion

This review identified 14 studies published over a 16-year period examining how electronic systems are used to collected PRO data after surgery. They were conducted in the USA and Europe, and mostly used commercial ePRO systems. These systems were often used in conjunction with other instruments, and therefore their use in future research and clinical applications should consider the challenges of implementing and interpreting results from multiple tools. None of the studies reported that the ePRO system was integrated with EHRs, despite the importance of this process to facilitate clinical acceptability and assimilation of ePRO data into routine clinical practice^17^. Further research is therefore needed to understand potential barriers to the integration of ePRO systems with existing clinical systems.

Less than one-third of studies reported that ePRO results were used directly to inform the clinical care of individual patients. There is interest in innovative uses of ePRO systems in routine care^8^^,^^17^. However, wider adoption of these systems is dependent partly on the availability of comparative evidence on their clinical effectiveness, such as from ongoing RCTs^2^^,^^3^^,^^36^ Most studies identified in this review were observational in design, with just two RCTs comparing electronic collection of PROs with other forms of data collection, such as e-mail or telephone. Therefore, further evidence from randomized trials is required to understand better the potential clinical benefits, barriers and unexpected adverse effects before wider adoption of this technology. Similarly, engagement and endorsement by clinicians and policy-makers is essential if ePRO systems are assimilated into standard clinical practice. In the present review, only one study reported on the economic impact of using an electronic system. To inform clinical practice and commissioning, future studies should also consider the financial value of ePRO systems, from the perspectives of the health system and patients.

This is the first systematic review specifically to investigate the use of electronic systems to collect PRO data after discharge from hospital following surgery. The findings were derived from a thorough search of five electronic databases. By including results from all study designs (randomized and non-randomized studies), a comprehensive review of research evidence was achieved. However, only articles in the English language were included. It is therefore possible that some relevant studies were omitted from the review, although the number of, and reasons for, excluded articles have been presented. This review reported on patient groups and specialties, such as orthopaedic surgery, but did not include detailed information about patients’ demographic and clinical characteristics. Future research should ensure that data on patient characteristics are collected and reported, to support identification of specific patient groups that engage (and conversely groups that engage less) with the use of ePRO systems.

Additionally, the results may be not be generalizable to healthcare systems across the world, as 8 of the 14 reviewed studies were conducted in the USA. No studies from Asia, Australia or Canada were included in the review, and so the findings may not accurately reflect current ePRO system use in research or clinical practice in those continents. For example, unpublished material (such as recently completed studies) was also excluded, which might have affected the conclusions drawn. The focus of this review was on ePRO systems used to collect data after patient discharge following surgery. Studies on day-case surgeries were excluded as the process and duration of recovery from surgery, and common postoperative symptoms and problems, are typically distinct to operations requiring a hospital stay. Consequently, studies reporting on relevant ePRO systems may have been omitted. None of the 14 reviewed studies reported technical or methodological challenges encountered in setting up or using ePRO systems. This information on lessons learned is important to guide future research. However, articles describing this information may be missing from this review as feasibility studies that did not describe and report on collected ePRO data were excluded.

Few studies have examined the use of electronic systems to collect PRO data after discharge following surgery, and very few of these were RCTs. Most studies used commercial software to collect ePRO data, but there was lack of integration with hospital clinical systems. Future research may wish to include studies with day-case surgery populations where relevant ePRO systems are used. Reporting of future studies should be comprehensive and transparent, to include difficulties related to the ePRO system that are encountered during study design or implementation, and any potential solutions. Studies should also state the name of the ePRO system software, the electronic devices used to collect ePRO data and, where non-validated or modified questionnaires are used, these should be described.

## Supplementary Material

zraa072_Supplementary_Data
