# Supplementary material for: Electronic collection of patient-reported outcomes following discharge after surgery: systematic review
Source: BJS Open. 2021 Mar 30;5(2):zraa072. doi: 10.1093/bjsopen/zraa072 (PMC8007587; doi:10.1093/bjsopen/zraa072)
Supplement: zraa072_Supplementary_Data [file zraa072_supplementary_data.zip › BJS5-2020-06-0105 Supplementary tables.docx]

**Supplementary Table 1. Example search strategy for OVID Medline**

| **#** | **Search term** |
| --- | --- |
|  | **Patient reported outcomes concept** |
| 1 | Patient Reported Outcome Measures/ |
| 2 | Patient Outcome Assessment/ |
| 3 | "patient reported".ti,ab,kw,hw. |
| 4 | (HR-PRO or HRPRO or HRQL or HRQoL or QL or QoL or "quality of life").ti,ab,kw,hw. |
| 5 | (health?index* or health?indices or health?profile* or health?status).ti,ab,kw,hw. |
| 6 | ((patient* or self or carer* or proxy or proxies or symptom* or side?effect* or distress or complication* or disability or function* or subjective or utilit* or well?being) adj (apprais* or report* or rate* or rating* or base* or assess* or relate* or alert* or care or monitor* or manage* or control or outcome* or index or indices or instrument* or measure* or questionnaire* or profile* or scale* or score* or status or survey*)).ti,ab,kw,hw. |
| 7 | 1 or 2 or 3 or 4 or 5 or 6 |
|  | **Electronic concept** |
| 8 | Electronics/ |
| 9 | Computers/ |
| 10 | Computer Communication Networks/ |
| 11 | Online Systems/ |
| 12 | Internet/ |
| 13 | Telemedicine/ |
| 14 | Medical Informatics/ |
| 15 | Software/ |
| 16 | Mobile Applications/ |
| 17 | Computers, Handheld/ |
| 18 | Smartphone/ |
| 19 | (electronic or digital or online or web* or web?based or "mobile?device" or eHealth or mHealth or "mobile?health" or "health information technology" or app).ti,ab,kw,hw. |
| 20 | 8 or 9 or 10 or 11 or 12 or 13 or 14 or 15 or 16 or 17 or 18 or 19 |
|  | **At home/after discharge concept** |
| 21 | Remote Consultation/ |
| 22 | Home Care Services/ |
| 23 | (remote* or home or post?discharge or "after discharge" or "following discharge" or "leave hospital").ti,ab,kw,hw. |
| 24 | 21 or 22 or 23 |
|  | **Surgery concept** |
| 25 | Surgical procedures, operative/ |
| 26 | (surgery or surgeries* or surgical or procedure* or operation* or intervention*).ti,ab,kw,hw. |
| 27 | 25 or 26 |
|  | **Combined concepts** |
| 28 | 7 and 20 |
| 29 | 24 and 28 |
| 30 | 27 and 29 |

**Supplementary Table 2. Summary of included studies (n=14)**

| **Study** | **Study country** | **Study design** | **Type of surgery** | **Sample size** | **Follow-up duration** | **Intervention** | **Primary outcome** | **Name of ePRO system** | **Frequency of ePRO data collection** |
| --- | --- | --- | --- | --- | --- | --- | --- | --- | --- |
| Begg et al, 2003^24^ | Scotland | Prospective cohort | Vaginal abdominal procedures and laparoscopies | 32 | 1 month | Electronic diary | Extent and duration of symptoms | Clinitrac | Twice a day |
| Bennett et al, 2016^25^ | USA | Randomised controlled trial | Colorectal cancer surgery | 435 | Other^a^ | Survey completion by web, telephone or interactive voice response system mode | Mode equivalence | Webcore | Daily |
| Bohu et al, 2016^26^ | France | Non-randomised trial | Shoulder stablisation | 46 | ≥1 year | Outpatient procedure | Need for inpatient care | Web Survey | Daily (day 0 and 1) then ≥1 year after surgery |
| Cnossen et al, 2014^27^ | The Netherlands | Non-randomised trial | Head and neck cancer surgery | 33 | 6 weeks | Head Matters - multimodal guided self-help exercise programme | Uptake, adherence and exercise performance | Not stated | Not stated |
| Cowan et al, 2016^28^ | USA | Prospective cohort | Laparotomy for gynaecological cancer | 91 | 6 weeks | Web-based PRO system and alerts to study nurses | Patient experience of system | Symptom Tracking and Reporting (STAR) system | Pre-surgery then weeks 3 and 6 after surgery |
| Davidovitch et al, 2018^29^ | USA | Retrospective cohort | Total hip arthroplasty | 268 | 12 weeks | Electronic patient rehabilitation application with home health service | Noninferiority in PROs | Force | Pre-surgery and at 12 weeks after surgery |
| Dias Correia et al, 2019^30^ | Portugal | Quasi-randomised study | Total hip arthroplasty | 66 | 6 months | Home-based digital rehabilitation programme | Patient uptake and safety of digital physiotherapy system | SWORD | ≥1 time a week |
| Lutz et al, 2016^31^ | France | Non-randomised trial | Anterior cruciate ligament (ACL) repair | 1076 | 5 days | Out-patient arthroscopic ACL repair | Pain intensity | Web Survey | Pre-surgery, evening after surgery, during night after surgery then 1, 3 and 5 days after surgery |
| McElroy et al, 2016^32^ | USA | Prospective cohort | Cardiac surgery | 443 | 30 days | Readmission Reduction Program plus Digital Health Kit | Postoperative readmission within 30 days of surgery | Digital Health Kit | Daily |
| Ramkumar et al, 2019^33^ | USA | Prospective cohort | Total knee arthroplasty | 25 | 12 weeks | Mobile app linked to knee sleeve | Frequency of data interruptions and patient acceptance | TKR | Weekly |
| Rosner et al, 2018^34^ | USA | Retrospective cohort | Hip, knee and shoulder arthroscopic procedures; knee arthrotomy | 371 | 90 days | N/A | Agreement between patient self-report of health care events and claim database records | Digital patient engagement (DPE) platform | According to procedure-specific care plan |
| Segura-Sampedro et al, 2017^35^ | Spain | Prospective cohort | Appendectomy | 15 | 7 days | Telemedicine follow-up | Safety and feasibility of telemedicine follow-up | Not stated | Once |
| Sun et al, 2017^36^ | USA | Prospective cohort | Gastrointestinal and hepato-pancreato-biliary cancer surgery | 20 | 2 weeks | Wireless monitoring of steps | Functional recovery (number of daily steps) | DatStat | 1-3 times a week |
| Mousa et al, 2019^37^ | USA | Randomised controlled trial | Vascular surgery | 30 | Other^b^ | TeleHealth Electronic Monitoring | 30-day readmissions and deep surgical site infections | Enform | Daily |

Key: ^a^Consecutive days (number of days not reported); ^b^Until first follow-up visit - no further detail provided
